# Maternal and Neonatal Serum Zinc Level and Its Relationship with Neural Tube Defects

**DOI:** 10.3329/jhpn.v28i4.6040

**Published:** 2010-08

**Authors:** Arjun Chandra Dey, Mohammod Shahidullah, Mohammad Abdul Mannan, Mohammad Khaled Noor, Laxmi Saha, Shahana A. Rahman

**Affiliations:** ^1^ Department of Neonatology, Bangabandhu Sheikh Mujib Medical University, Shahbagh, Dhaka 1000, Bangladesh; ^2^ Department of Obstetrics and Gynaecology, Dhaka Medical College Hospital, Dhaka 1000, Bangladesh; ^3^ Department of Paediatrics, Bangabandhu Sheikh Mujib Medical University, Shahbagh, Dhaka 1000, Bangladesh

**Keywords:** Atomic absorption spectrophotometer, Case-control studies, Neural tube defects, Serum zinc, Zinc deficiency, Bangladesh

## Abstract

Neural tube defect (NTD) is a multi-factorial disorder in which nutritional, genetic and environmental factors are involved. Among the nutritional factors, low level of serum zinc has been reported from different parts of the world. This hospital-based case-control study was conducted with the objective of finding the relationship between serum zinc level in newborns and their mothers and NTDs in a Bangladeshi population. The study was conducted during August 2006–July 2007 at the Bangabandhu Sheikh Mujib Medical University (BSMMU) in Dhaka. In total, 32 mothers and their newborns with NTDs were included as cases and another 32 mothers with their normal babies were included as controls. Concentration of serum zinc was determined by pyro-coated graphite furnace atomic absorption spectrophotometer (GF-AAS). The mean age of the case and control mothers was 25.28 years and 24.34 years respectively. The mean gestational age of the case newborns was 36.59 weeks and that of the control newborns was 37.75 weeks. The mean serum zinc level of the case and control mothers was 610.2 μg/L and 883.0 μg/L respectively (p<0.01). The mean serum zinc level of the case and control newborns was 723 μg/L and 1,046 μg/L respectively (p<0.01). In both case and control groups, the serum zinc level of the newborns positively correlated with that of the mothers. The serum zinc levels of the mothers and newborns negatively correlated with NTDs. Mothers with serum zinc level lower than normal were 7.66 [95% confidence interval (CI) 2.5-23.28] times more likely to have NTDs compared to the normal zinc level of mothers. After adjusting for the zinc level of the newborns, parity, and age of the mothers, this risk reduced 1.61 times [confidence interval (CI) 95% 0.24-8.77]. On the other hand, the low serum zinc level of the newborns was 7.22 times more associated with NTDs compared to the newborns with the normal serum zinc level, which was statistically significant (p=0.001). After adjusting for other factors, such as maternal age and parity, newborns with the low serum zinc level was found to be 9.186 times more likely to be associated with NTDs compared to newborns with normal serum zinc level. Based on the findings, it may be concluded that the low serum zinc levels of newborns may be associated with NTDs. To confirm these findings, a further study with a larger sample-size is recommended. Moreover, a follow-up study with zinc supplementation to pregnant women and its impact on NTDs is also recommended.

## INTRODUCTION

Neural tube defects (NTDs) account for most congenital anomalies of the central nervous system (CNS). NTDs are a group of very serious birth-defects that arise when the neural tube fails to develop into the brain and spinal cord during the first month of pregnancy.

The CNS appears at the beginning of the third week of pregnancy as a slipper-shaped plate of thickened ectoderm—the neural plate—in the mid-dorsal region in front of the primitive node. Its lateral edges soon elevate to form the neural folds (1). The neural folds elevate, approximate each other, and finally close to form the neural tube.

The nutritional status of pregnant women may influence the vulnerability to NTDs in the foetus. Folate deficiency is a well-known cause of NTDs. The search for other aetiological factors, particularly nutritional factors, has been continuing. In recent years, zinc and vitamin B12 have attracted the attention of researchers in relation to the development of NTD.

Zinc is essential for the growth and development of the foetus and plays a critical role in many cellular reactions, including gene transcription and cell division and differentiation. The inadequate intake of zinc is associated with NTDs in both animals and humans (2). The essentiality of zinc in the formation of neural tube is further supported by the observation that women with acrodermatitis enteropathica, a disorder of impaired zinc absorption from the intestine, are at high risk for babies with NTDs (3), although the possible mechanism is not known. Women with second trimester-induced abortion, resulting from NTDs, have a significant low level of serum zinc and selenium (4). Authors of several studies recommended supplementation of zinc, in addition to folic acid, for the further decrease in the recurrence and occurrence of NTDs (2–4). However, others found increased zinc content in umbilical cord serum in anencephalic newborns and spina bifida-affected newborns (5). Simultaneously in mothers having babies with NTD, total serum zinc was similar to controls but there was a shift in the distribution of zinc from alpha-2 macroglobulin to albumin. Foetal hyperzincaemia and elevated maternal albumin-bound zinc found in the study suggest that the NTD-foetus receives zinc but does not normally uses it.

Newborns with NTDs have a significantly low level of serum zinc, supporting zinc deficiency as an association of NTDs (6,7). Zeyrek *et al*. reported that low maternal zinc and high copper during pregnancy may be responsible for NTDs (8).

The worldwide incidence of NTDs is estimated to be around 1 per 1,000 livebirths (9). However, there are remarkable variations in the incidence of NTDs and other CNS defects. In certain regions of China, the incidence of NTD is 1 in 100 livebirths, being the highest (10) whereas, in the Scandinavian countries, it is only 1 in 5,000 livebirths (7). In the United States, the incidence of NTDs was estimated at 1 per 1,000 deliveries, anencephaly at 0.6-0.8 per 1,000 livebirths, and open spina bifida as 0.5-0.8 per 1,000 livebirths (11). No data are available on the incidence and prevalence of NTDs in Bangladesh, although this malformation is fairly common in the clinical practices.

This study was designed to see the correlation of NTDs with maternal and newborn serum zinc concomitantly.

## MATERIALS AND METHODS

This case-control study was conducted at the Department of Neonatology, Bangabandhu Sheikh Mujib Medical University (BSMMU) Hospital in Dhaka, during August 2006–July 2007. Cases were selected from the Departments of Neonatology, Obstetrics-Gynaecology, and Neurosurgery, BSMMU, Dhaka Medical College Hospital (DMCH), and Maternal and Child Health Training Institute (MCHTI), Azimpur, Dhaka.

Of the three hospitals, the BSMMU primarily deals with cases referred from all over the country, which logically is not representative of the general population. The DMCH is more easily accessible for the general people from all over the country and, hence, is representative of the population. The MCHTI, a referral centre for obstetric patients, conducts a good number of deliveries per year.

NTDs were diagnosed on ultrasonography in the later weeks of pregnancy and passively on referral after birth. Alpha fetoprotein level, a good indicator of NTD, was not assayed as most mothers were not capable financially, and this is also not an easily-available investigation in the country.

### Study population

Newborn babies with NTDs and their mothers were considered cases whereas normal babies and their mothers were included as controls. The study excluded mothers taking zinc supplement during anytime of pregnancy and newborn babies with other major congenital anomalies and syndrome in babies where NTD is a component, e.g. Meckel Gruber syndrome. Controls were randomly selected. Blood samples were drawn from the mothers and their babies within one week after delivery.

### Sample-size

The sample-size was calculated using the following formula:

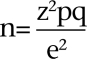

where z=1.96, p=1 (worldwide incidence 1 in 1,000 livebirths, q=100-p, e=5, acceptable error).

The formula dictates that the sample-size for the study would be around 15. However, given the one-year timeframe of the study (the study was done as a thesis for MD in neonatology) and also availability of cases in the three hospitals, the sample-size was fixed at 30. During the study period, two more cases met the inclusion criteria. Therefore, in total, 32 cases (mothers and babies with NTDs) were enrolled. However, two newborns with anencephaly were stillborn, and one died immediately after birth. As the serum zinc of those three newborns could not be measured, serum zinc was measured in 29 pairs of mother and baby and three mothers without babies. Controls were limited to 32 pairs mainly due to financial constraints.

### Data-collection method

A preformed data-collection sheet was used for collecting data. Baseline information was collected from cases who agreed to participate in the study. Body mass index (BMI) of the mothers was recorded. Emphasis was given on measuring the occipito-frontal circumference while examining the newborn with NTD to exclude the possible association of hydrocephalus.

### Biochemical analysis

Plastic wares, free of metallic contamination, especially zinc, were used for collecting blood specimens. The plastic wares were kept immersed in detergent water at least for half an hour, washed thoroughly with running water, and allowed to dry in air. The plastic wares were then kept immersed for 24 hours in 20% nitric acid. After 24 hours, all the equipment were washed three times in de-ionized water and were air-dried. The air-dried containers were stored in a capped plastic container, to be used later in sample collection. Using a disposable plastic syringe, taking aseptic precautions, 5 mL of venous blood was collected from the mothers and newborns within seven days after delivery. It was then transferred to a de-ionized plastic test-tube. Blood was centrifuged at 3,500 rpm for five minutes, and the serum was transferred to a clean polypropylene tube. The sample was then frozen at -35 °C for long-term storage until assay for zinc concentrations was done. Serum zinc was estimated in the Department of Biochemistry, BSMMU, using graphite furnace atomic absorption spectrophotometer (GF-AAS, 6650 Shimadzu). The level of serum zinc was expressed in μg/L.

### Statistical analyses

Data were analyzed using the SPSS software for Windows (version 13.0) (SPSS Inc., Chicago, IL). Chi-square test was used for comparing proportions. Student's unpaired *t*-test was applied for testing the differences between continuous variables. Cross-tabulation and multivariate analyses were used for exposing the associations between the dependent and the independent variables. A two-sided p value of <0.05 was considered significant at 95% level. Pearson's correlation test was used for seeing the correlation between continuous variables. However, the comparison between a quantitative variable and a qualitative variable was made with the help of Point biserial correlation.

### Ethics

Before the study, permission was taken from the Chairmen and Department Heads of the respective institutions. The Ethical Committee of the Department of Paediatrics, BSMMU, approved the protocol. Finally, informed written consent was taken from all the mothers/parents/guardians after full explanations of the nature and purpose of the procedure used for the study. Anonymity was maintained throughout the study, and none of the names was used in the database.

## RESULTS

In the one-year study period in the DMCH, 10,861 deliveries were conducted, of which 16 cases (0.1%) had NTDs. Of 5,778 deliveries in the BSMMU, 11 cases (0.2%) had NTDs, and of 3,628 deliveries in the MCHTI, Azimpur, one case had NTD. The incidence of NTDs, on average, was 1.3 per 1,000 livebirths. Four cases were delivered either in the home or in other hospitals and were later referred to one of these hospitals.

Table 1 shows the baseline characteristics of the affected mothers. Age, BMI, occupation, socioeconomic condition, and status of antenatal check-ups were compared between the case and the control mothers but no significant difference was observed.

**Table 1.** Variables analyzed in the study

Variable	Case	Control	p value
Age (years) of mothers (mean±SD)	25.3±6.1	24.3±5.03	0.505
Age-group (years) of mothers (%)			
17-24	56.3	65.6	
25-32	31.3	25.0
33-40	12.5	09.4	
BMI (mean±SD)	21.8±2.3	22.7±3.2	0.168
Occupation of mothers			
Housewife, no. (%)	32 (100)	30 (93.8)	
Employed, no. (%)	0 (0)	2 (6.3)	0.472
Socioeconomic status			
Middle*, no. (%)	13 (40.6)	11(34.4)	
Lower†, no. (%)	19 (59.4)	21 (65.6)	0.565
Parity of mothers (mean±SD)	2.2±1.1	1.5±0.8	0.007
Antenatal check-up			
Regular$, no. (%)	14 (43.7)	8 (25.0)	
Irregular§, no. (%)	8 (25.0)	5 (15.6)	
None (%)	10 (31.3)	19 (59.4)	0.077
Gestational age at delivery (mean±SD)	36.6±1.8	37.8±1.2	0.004
Mode of delivery			
Vaginal delivary, no. (%)	24 (75.0)	15 (46.9)	
Caesarean section, no. (%)	8 (25.0)	17 (53.1)	0.021
Volume of liquor			
Normal	29 (90.6)	32 (100)	
High	3 (9.4)	0 (0.0)	0.237

The p value is significant if <0.05;

*Middle class was categorized arbitrarily based on family income (Tk 10,000-20,000 per month);

†Lower class was categorized arbitrarily based on family income (<Tk 10,000 per month);

$At least 3 visits during pregnancy;

§Irregular antenatal check-up: less than 3 visits during pregnancy;

BMI=Body mass index;

SD=Standard deviation

The mean±standard deviation (SD) parity of the mothers of newborns with and without NTDs was 2.2±1.1 and 1.5±0.8 respectively**.** The difference was significant (p<0.01). The mean±SD gestational age at delivery of the cases was 36.6±1.8 weeks and that of the controls was 37.8±1.2 weeks. The difference was significant (p<0.01).

The large majority (75%) of the case mothers delivered their NTD-affected babies per vagina whereas the caesarian section rate was higher among the controls. The difference was significant (p<0.05).

Various types of NTDs were observed among the study subjects (Table 2). Of the 32 cases, 20 were meningomyelocele (62.5%), followed by encephalocele (n=7, 21.8%), anencephaly (n=3, 9.4%), and others (n=2, 6.2%). Eighty-five percent of the meningomyelocele cases were associated with hydrocephalus.

**Table 2.** Types of NTD among cases studied (n=32)

Type of NTD	No. of cases	%	Associated hydrocephalus	
			No.	%
Meningomyelocele	20	62. 5	17	85.0
Encephalocele	7	21.9	0	0
Anencephaly	3	9.4	0	0
Meningocele	1	3.1	0	0
Lipomeningocele	1	3.1	0	0
Total	32	100	17	53.1

NTD=Neural tube defect

The mean±SD concentration of maternal serum zinc level in the case group was 610.2±53.1 μg/L and that in the control group was 883.0±65.2 μg/L (Table 3). The difference was significant (p<0.01) between the two groups. The mean±SD serum zinc level of the newborns in the cases and controls was 723.0±56.3 and 1,046.0±56.3 μg/L respectively (Table 3). The difference was significant (p<0.01).

**Table 3.** Serum zinc level of mothers and newborns in case and control groups

Group	Zinc level (μg/L)		p value
	Mean±SD	Range	
Mothers			
Case (n=32)	610.2±53.1	135.0-1,610.0	0.002
Control (n=32)	883.0±65.2	435.0-1,912.0	
Newborns			
Case (n=29)	723.0±56.3	345.0-1,710.0	0.001
Control (n=32)	1,046.0±56.3	621.0-2,014.0	

SD=Standard deviation

**Fig. 1.**
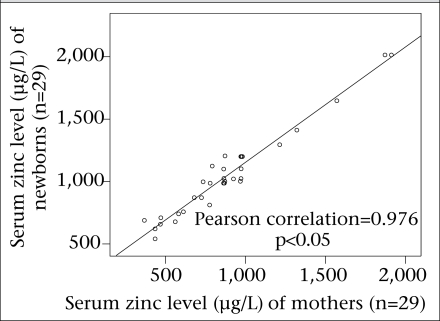
Correlation of serum zinc level of control mother and newborns

**Fig. 2.**
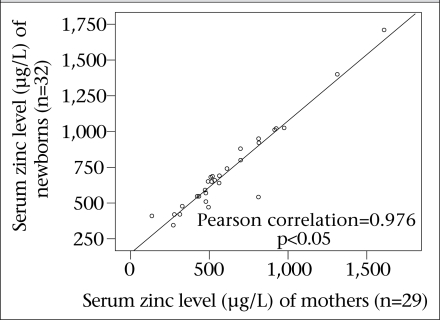
Correlation of serum zinc level of case mothers and newborns

The maternal and newborn serum zinc levels positively correlated with each other (R=0.976, p<0.05), which means that the serum level of the mothers and newborns in both case and control groups had a linear relationship (Fig. 1 and 2).

**Fig. 3.**
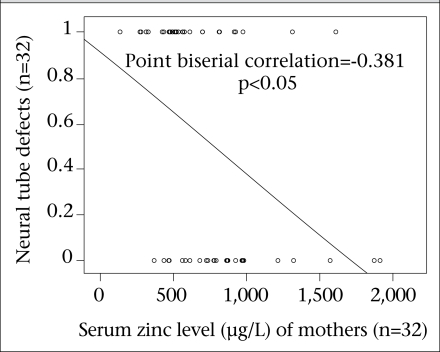
Correlation between maternal serum zinc level and neural tube defects

**Fig. 4.**
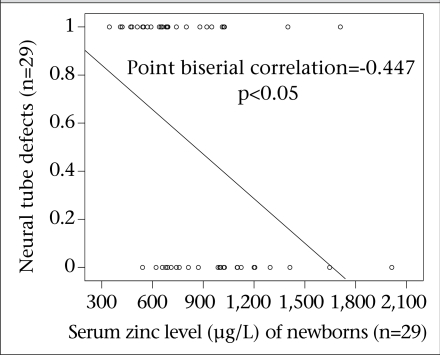
Correlation between newborn serum zinc level and neural tube defects

The results showed a negative correlation between maternal serum zinc level and NTDs (Fig. 3). Similarly, a negative correlation was also observed between the serum zinc level of the newborns and NTDs (Fig. 4). This means that the newborn babies with the highest level of serum zinc had the lowest number of NTDs and vice versa. This correlation was significant.

Multiple logistic regression analysis was done where different variables, such as maternal zinc level (Model 1), neonatal zinc level (Model 2), maternal parity (Model 3), and maternal age (Model 4) were analyzed as risk factors of NTDs (Table 4). Model 1 showed that the unadjusted odds ratios, i.e. mothers with low serum zinc level (<700 μg/L), were significantly associated with NTD-affected babies [odds ratio (OR)=7.7, 95% confidence interval (CI) 2.5-23.3]. Model 2 showed that the newborns with the low serum zinc level were strongly associated with NTDs compared to those who had normal serum zinc (OR=7.2, 95% CI 1.4-38.74). It was observed in Model 3 that multiparity had no significant relationship with NTDs, and it slightly lowered the level of significance of the impact of the newborn zinc level on NTDs. Maternal age had no significant effect on NTDs but, after adjusting for maternal age and parity, the impact of the newborn zinc level on NTDs became higher (OR=9.2, 95% CI 1.5-56.9). This means that, after adjusting for other variables, the newborns with the low serum level were significantly (p<0.05) associated with the occurrence of NTDs.

**Table 4.** Logistic regression analysis of variables

Indicator	Model 1	Model 2	Model 3	Model 4
	Unadjusted OR (CI)	Adjusted OR (CI)	Adjusted OR (CI)	Adjusted OR (CI)
Zinc level of mothers >700† (0–700)	7.7* (2.5-23.3)	1.9 (0.4-9.8)	1.9 (0.3-9.9)	1.6 (0.3-8.6)
Zinc level of newborns >700† (0–700)		7.2* (1.3-38.7)	6.8* (1.2-37.0)	9.2* (1.5-56.9)
Multiparity				
Primi†			1.6 (0.19-12.4)	1.5 (0.1-25.8)
Multipara				
Age (years)				
17-24†				
25-33				2.1 (0.51-5.8)
34-40				0.9 (0.1-12.4)

†Reference category;

*Significant (p<0.05);

CI=Confidence interval;

OR=Odds ratio

## DISCUSSION

Our study found that the mean age of the case mothers was 25.3 years and that of the control group was 24.3 years, which was comparable with the mean age of mothers in a study in Iran (7). It was found in another study that the incidence of NTDs decreased with increased maternal age (12). On the other hand, a U-shaped distribution of NTDs in relation to age has been described (13,14). Our study did not find any correlation of this kind, which may be attributable to the relatively smaller sample-size.

Higher parity was an important factor relating to NTDs in the present study as the multipara mothers were affected significantly more than the primipara mothers. Literature suggests a variable pattern of findings. Results of a meta-analysis showed that pregnancy with higher parity was more likely to have a spina bifida defect than with lower parity (15). On the other hand, a striking contrast was also observed where risk of spina bifida decreased with parity (16).

In the present study, the mean gestational age of the case group was 36.6 weeks whereas that of the control group was 37.8 weeks. This finding correlates with the well-established fact that any congenital malformation in the foetus may lead to preterm delivery. NTD is particularly known to be associated with preterm delivery (16).

Anencephaly is a well-known cause of polyhydramnios (17,18). In our study, three cases of anencephaly were associated with polyhydramnios.

Worldwide, meningomyelocele is most common among NTDs and is associated with hydrocephalus in more than 85% of cases. Results of studies showed that meningomyelocele contributed 86.8% of spina bifida cystica cases (11,19), which is consistent with the findings of the present study. In the present study, about 63% of the NTDs were meningomyelocele, of which 85% were associated with hydrocephalus.

The results of the present study showed that maternal zinc deficiency was associated with NTDs. Some studies found a similar correlation between zinc deficiency and NTDs (6,20). Zeyrek *et al*. reported a maternal low serum zinc level and a high copper level as an association with NTDs (8). A recent study in Iran reported maternal zinc deficiency to be associated with NTDs (21). However, results of some studies also suggest that zinc deficiency was not related to NTDs (22,23), although population-based case-control studies concluded that the risk of NTDs in infants and foetuses decreased with increasing maternal zinc intake (24,25).

The NTD-affected newborns had a lower level of serum zinc (mean 723 μg/L) compared to the unaffected newborns (mean 1,046 μg/L), suggesting that zinc deficiency is associated with NTDs. The association of the lowest serum zinc in the newborns and NDTs is quite consistent with that of a study in Iran (7). Results of a study in Turkey showed newborn serum zinc and selenium to be low in NTDs compared to normal controls (26). An Indian study that evaluated maternal and newborn hair zinc levels in association with NTDs found that the hair zinc levels in both mothers and newborns were significantly low compared to normal controls and recommended a follow-up study with zinc supplementation in the periconceptional period and outcome in the subsequent pregnancy (27).

Multiple logistic regression analysis in our study revealed that an unadjusted maternal low zinc level was associated with NTDs but it was negatively confounded by other variables, such as serum zinc level of newborns, age of mothers, and parity. The low serum zinc levels of the newborns were significantly associated with NTDs compared to those having the normal serum zinc levels. When adjusted with age of mother and parity, the low serum zinc level of the newborns was more significantly associated with NTDs.

### Limitations

The sample-size in the study was small, and blood samples from the newborns were drawn within one week, not immediately after birth, which would have generated a better understanding about the serum zinc status of the newborns. Moreover, some other known risk factors, such as diabetes mellitus and exposure to arsenic, were not considered while selecting the cases. The serum zinc level of the mothers during the first trimester was not known which is crucial for the development of NTDs. Moreover, it was not known whether the zinc level after birth was reflective of that in the first trimester. As NTD is an uncommon disorder, the findings of the present study would be more valid if the control sample-size was a larger one.

### Conclusions

The results of the present study suggest, among the factors evaluated, that the low serum zinc level in newborns is significantly associated with NTDs. The low level of maternal serum zinc seemed to be associated but it was confounded negatively by other variables.

A further study with a larger sample-size and a control from the representative group of population would yield a more acceptable finding in this regard. Moreover, a randomized trial with periconceptional zinc supplementation and its impact on subsequent pregnancy with respect to NTDs may generate more conclusive evidence of the relationship between the serum zinc level and NTDs.

## ACKNOWLEDGEMENTS

The authors thank all doctors and nurses of the Department of Neonatology, BSMMU, for their constant cooperation and inspiration. The authors also thank Prof. Afzal Hossain, Chairman, Department of Neurosurgery, BSMMU; Prof Sultana Jahan, Chairperson, Department of Obstetrics and Gynaecology, BSMMU; Prof. Abid Hossain Mollah, Head of Paediatrics, Dhaka Medical College Hospital (DMCH); Prof. Kohinur Begum, Head of Obstetrics-Gyenaecology, DMCH; Prof. Sader Hossain Chowdhury, Head, Department of Neurosurgery, DMCH; and Dr. Roksana Ivy, Consultant of obstetrics and Gynaecology, MCHTI, Dhaka, for their cooperation in selecting cases from their respective institutions. The authors gratefully acknowledge Prof. Iqbal Arslan, Chairman, Department of Biochemistry, Dr. Rizwanur Rahman, MD thesis-part student, and Dr. Waliur Rahman, Medical Officer, Department of Biochemistry, BSMMU, for their active support in the assay of serum zinc. Special thanks are also due to Dr. Soumitra Sarker, Assistant Professor of Neurosurgery, Mymensingh Medical College and Hospital, for his support and encouragement. Finally, the authors appreciate and acknowledge Prof. Syeda Afroza, Head of Paediatrics, Shahid Suhrawardy Medical College and Hospital, Dhaka and Prof. Manjare Shamim, Chairman, Department of Anatomy, BSMMU, for their critical reading of the write-up of this research work.

## References

[1] Sadler TW (2006). Development of nervous system. Langman's Medical embryology, 10^th^ ed..

[2] Tamura T, Goldenberg RL (1996). Zinc nutriture and pregnancy outcome. Nutr Res.

[3] Mambidge KM, Neldner KH, Walravens PA (1975). Zinc, acrodermatitis enteropathica and congenital malformations. Lancet.

[4] Cengiz B, Söylemez F, Oztürk E, Cavdar AO (2004). Serum zinc, selenium, copper, and lead levels in women with second-trimester induced abortion resulting from neural tube defects: a preliminary study. Biol Trace Elem Res.

[5] Zimmerman AW (1984). Hyperzincemia in anencephaly and spina bifida: a clue to the pathogenesis of neural tube defects?. Neurology.

[6] Carrillo-Ponce Mde L, Martínez-Ordaz VA, Velasco-Rodríguez VM, Hernández-García A, Hernández-Serrano MC, Sanmiguel F (2004). Serum lead, cadmium, and zinc levels in newborns with neural tube defects from a polluted zone in Mexico. Reprod Toxicol.

[7] Golalipour MJ, Mansourian AR, Keshtkar A (2006). Serum zinc levels in newborns with neural tube defects. Indian Pediatr.

[8] Zeyrek D, Soran M, Cakmak A, Kocyigit A, Iscen A (2009). Serum copper and zinc levels in mothers and cord blood of their newborn infants with neural tube defects: a case-control study. Indian Pediatr.

[9] Zupancic JAF, Cloherty JP, Eichenwald EC, Stark AR (2004). Neural tube defects. Manual of neonatal care, 5^th^ ed..

[10] Xiao KZ (1989). [Epidemiology of neural tube defects in China]. Zhonghua Yi Xue Za Zhi.

[11] Weindling AM, Rennie JM, Rennie JM (2005). Neurological problems of the neonate: central nervous system malformation. Roberton's Textbook of neonatology.

[12] McDonnell RJ, Johnson Z, Delaney V, Dack P (1999). East Ireland 1980–1994: epidemiology of neural tube defects. J Epidemiol Community Health.

[13] Hendricks KA, Simpson JS, Larsen RD (1999). Neural tube defects along the Texas-Maxico border, 1993–1995. Am J Epidemiol.

[14] Whiteman D, Murphy M, Hey K, O'Donnell M, Goldacre M (2000). Reproductive factors, subfertility, and risk of neural tube defects: a case-control study based on the Oxford Record Linkage Study Register. Am J Epidemiol.

[15] Vieira AR (2004). Birth order and neural tube defects: a reappraisal. J NeurolSci.

[16] Illinois Department of Public Health (2004). Division of Epidemiologic Studies. Prevalence of neural tube defects in Illinois 1989–2002.

[17] Goldstein RB, Filly RA (1988). Perinatal diagnosis of anencephaly: spectrum of sonographic appearance and distribution from the amniotic band syndrome. Am J Roentgenol.

[18] Lipitz S, Meizner I, Yagel S, Shapiro I, Achiron R, Schiff E (1995). Expectant management of twin pregnancies discordant for anencephaly. Obstet Gynecol.

[19] Alatise OI, Adeolu AA, Komolafe EO, Adejuyigbe O, Sowande OA (2006). Pattern and factors affecting management outcome of spina bifida cystica in lle-lfa, Nigeria. Pediatr Neurosurg.

[20] Hinks LJ, Ogilvy-Stuart A, Hambidge KM, Walker V (1989). Maternal zinc and selenium status in pregnancies with neural tube defects. Br J Obstet Gynaecol.

[21] Golalipour MJ, Vakili MA, Mansourian AR, Mobasheri E (2009). Maternal serum zinc deficiency in cases of neural tube defect in Gorgan, north Islamic Republic of Iran. East Mediterr Health J.

[22] Hambridge M, Hackshaw A, Wald N (1993). Neural tubedefects and serum zinc. Br J Obstet Gynaecol.

[23] Milunsky A, Morris JS, Jick H, Rothman KJ, Ulcickas M, Jick SS (1992). Maternal zinc and fetal neural tube defects. Teratology.

[24] Shaw GM, Todoroff K, Schaffer DM, Selvin S (1999). Periconceptional nutrient intake and risk for neural tube defect-affected pregnancies. Epidemiology.

[25] Velie EM, Block G, Shaw GM, Samuels SJ, Schaffer DM, Kulldorff M (1999). Maternal supplemental and dietary zinc intake and the occurrence of neural tube defects in California. Am J Epidemiol.

[26] Karatas F, Aygun D, Gurusu F Serum zinc and selenium levels in mother and their newborns with neural tube defects (abstract) CABI abstracts.

[27] Srinivas M, Gupta DK, Rathi SS, Grover JK, Vats V, Sharma JD (2001). Association between lower hair zinc levels and neural tube defects. Indian J Pediatr.

